# Surface passivation of semiconducting oxides by self-assembled nanoparticles

**DOI:** 10.1038/srep18449

**Published:** 2016-01-13

**Authors:** Dae-Sung Park, Haiyuan Wang, Sepehr K. Vasheghani Farahani, Marc Walker, Akash Bhatnagar, Djelloul Seghier, Chel-Jong Choi, Jie-Hun Kang, Chris F. McConville

**Affiliations:** 1 Department of Physics, University of Warwick, Coventry, CV4 7AL, United Kingdom; 2 Science Institute, University of Iceland, Dunhaga 3, Reykjavik, IS-107, Iceland; 3 School of Semiconductor and Chemical Engineering, Chonbuk National University, Jeonju, 561-756, Republic of Korea; 4 Department of Nano and Electronic Physics, Kookmin University, Seoul, 136-702, Republic of Korea

## Abstract

Physiochemical interactions which occur at the surfaces of oxide materials can significantly impair their performance in many device applications. As a result, surface passivation of oxide materials has been attempted via several deposition methods and with a number of different inert materials. Here, we demonstrate a novel approach to passivate the surface of a versatile semiconducting oxide, zinc oxide (ZnO), evoking a self-assembly methodology. This is achieved via thermodynamic phase transformation, to passivate the surface of ZnO thin films with BeO nanoparticles. Our unique approach involves the use of Be_*x*_Zn_1-*x*_O (BZO) alloy as a starting material that ultimately yields the required coverage of secondary phase BeO nanoparticles, and prevents thermally-induced lattice dissociation and defect-mediated chemisorption, which are undesirable features observed at the surface of undoped ZnO. This approach to surface passivation will allow the use of semiconducting oxides in a variety of different electronic applications, while maintaining the inherent properties of the materials.

For the next generation of optoelectronic devices, oxide-based applications such as transparent electronic circuitry have recently received a great deal of interest. These materials have high optical transparency, excellent thermal stability and electrical characteristics often under severe environmental conditions^1^,^2^. Various wide band gap oxide semiconductors such as doped and undoped In_2_O_3_, SnO_2_, TiO_2_, and ZnO and their related alloys have been all extensively employed as integrated materials in many transparent optoelectronic applications^3^,^4^,^5^,^6^. However, the intrinsic physiochemical properties of these materials are strongly influenced by surface effects associated with variable composition, stoichiometry, mechanical stress, and chemical interactions with environmental species^7^,^8^,^9^. Thermal vulnerability of oxide materials also results in atomic diffusion, lattice distortions, and the subsequent formation of charged defects under thermodynamic equilibrium^10^,^11^. Crucially, however, interactions between environmental species and the oxide surface are facilitated by chemisorption. This leads to structural rearrangements of the cation and anion sites and differing charge distribution states across the oxide surface. Such variations in the ionic positions and charge densities can create additional surface states, which vary the intrinsic semiconducting characteristics (*e.g.* uniformity of carrier density and carrier transport) and limit the high-performance of oxide-based optoelectronics such as metal-oxide-based memory devices^12^,^13^,^14^. Hence, successful passivation of the surfaces of oxide materials to protect against thermal degradation and physiochemical coupling with ambient gas molecules is essential for the design of inert surfaces in device architecture^15^,^16^.

Phase transformations in metastable oxides and their mismatched heterostructures can modify initial material properties due to the impact on macroscopic atomic diffusion, mechanical, thermal, and other intrinsic properties of the oxides^17^,^18^,^19^. These transformations occur by the minimization of the Gibbs free energy in the phase diagram, which is driven by overcoming an energy barrier under pressure and temperature^20^,^21^. This leads to the formation of new phases, secondary crystallization or mixed phases and/or spinodal decomposition^22^,^23^. In the former case, constituent atoms diffuse towards either a surface or interface, resulting in the nucleation of a secondary phase due to the difference in partial molar free energy between the diffused/segregated solute and a solid phase under supersaturation^24^,^25^. When this occurs, particles nucleate and grow preferentially at energetically favourable sites, *e.g.*, non-equilibrium sites such as point defects, defect clusters, dislocations, and grain boundaries, thus rendering lower free energy by the crystal nucleation^26^,^27^. Interactions between any new phase and the surface of a thin film/nanocrystal can significantly influence the thermodynamics and kinetics of nucleation-and-growth, as well as the physical and chemical properties of the resulting particles and surfaces.

Understanding such phase transition phenomena and the growth kinetics of these secondary phase particles in various metal oxides is essential to control their functionalities (*e.g.* the size dependence for the control of surface-to-volume ratio of particles and an effective distribution of particles of different radii)^28^,^29^,^30^. Moreover, with particle coarsening features, *e.g.*, combined/competitive growth stages between the nucleation and curvature-induced coarsening of these nanoparticles, effective methods have been developed to fabricate the desired nanocrystals with uniform size distributions^31^,^32^,^33^,^34^. This enables the design of novel nanostructures with distinct chemical and physical properties with potential applications, for example, in nanoelectronics and energy storage media.

Here, we report a novel approach to the passivation of reactive oxide surfaces by spontaneous formation of functional secondary phase nanoparticles (NPs). This is achieved as a result of a phase transformation on a thermally-recrystallized metastable oxide. Using the example of beryllium zinc oxide (BZO), we present the detailed kinetic mechanisms found in the alloy. This is as a result of the surface Be concentration dependence on the nucleation-and-growth and the size-distribution of NPs. The functionalities of the NPs include passivation against thermal dissociation and surface chemisorption, are detailed experimentally and theoretically. The thermodynamic characteristics of the constituent atoms and physiochemical reactions at the surface of the transformed BZO observed experimentally have also been simulated by density functional theory (DFT) calculations of the atomic compensation and relaxation energies at model metastable surfaces.

## Results and Discussion

### The formation of secondary phase NPs at the surface of the BZO films

The BZO alloy films composed of different Be concentrations, *x* = 0.02 and 0.06, were grown on Al_2_O_3_(0001) substrates, and are signified as BZO(0.02) and BZO(0.06). The Be-concentration-dependent lattice shrinkage and band gap widening were observed in the as-grown BZO films as result of a chemical alloying of ZnO and BeO. To investigate the thermodynamics of the metastable BZO thin films, the strain relaxation and the subsequent phase transformations in the alloy films were systematically increased by varying the post-annealing temperatures, from *T*_A_ = 600 °C − 950 °C. Details of the film preparation and annealing conditions are given in Methods. The annealed BZO films show opposing trends between a decrease in the optical band gap energy and an increase in the surface Be concentration with increasing *T*_A_. These effects are indicative of transient out-diffusion of Be from the bulk to the surface of the metastable film through thermally increased recrystallization, and a lowering of the Be composition in the bulk (Supplementary Information 1 & 2).

**Figure 1:**
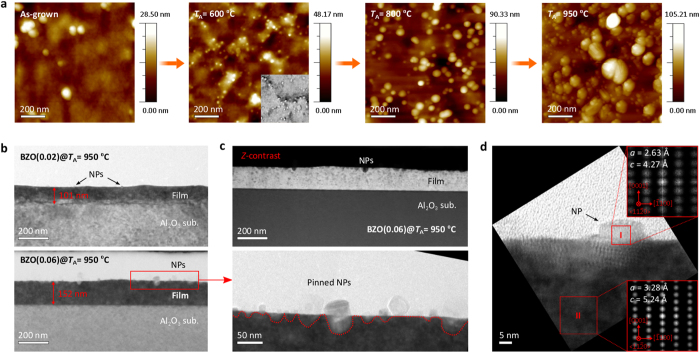
Morphological evolution of BeO NPs at the surface of transformed BZO films. (**a**) AFM topography (1 × 1 μm^2^) images for the as-grown and annealed BZO(0.02) films (*T*_A_ = 600 − 950 °C). The inset is a phase image (0.5 × 0.5 μm^2^) of the film annealed at *T*_A_ = 600 °C. (**b**) Cross-sectional TEM images of the BZO(0.02 and 0.06) films annealed at *T*_A_ = 950 °C. The thickness of the films was determined to be 101 ± 12 nm and 152 ± 8 nm, respectively. (**c**) The top image is a *Z*-contrast dark field STEM image for the annealed BZO(0.06) film. The lower image is a magnified TEM image for the surface of the film corresponding to the red rectangular in the bottom image of (**b**). The red-dot line is a guide to the eye for the transparent NPs at the surface. (**d**) High resolution TEM image along with the 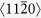 zone-axis at the surface of the annealed BZO(0.06) film. The right-top and right-bottom insets are Fast-Fourier transforms (FFTs), which correspond to the red-square areas, I and II, inside the surface NP and the film layers underneath, respectively. The FFTs were filtered masking 0001 and 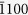 reflections. The calculated *a*- and *c*-lattice parameters for each area, obtained by extracting the plane spacings, were denoted in the insets.

On the basis of this transient atomic redistribution and strain relaxation along with the phase transformation of the BZO films, variations in the surface morphology with *T*_A_ were investigated. Figure 1a shows the AFM topographic images of the structural evolution at the surface of the BZO(0.02) films as a function of *T*_A_. The formation of NPs can clearly be seen with higher density near grain boundaries for *T*_A_ = 600 °C (see inset for the phase image, 0.5 × 0.5 μm^2^). This is because mass transport mainly occurs along the grain boundaries and atomically imperfect defects are active nucleation sites^35^,^36^. The surface NPs migrate towards larger ones at *T*_A_ ≥ 800 °C due to Ostwald ripening (OR), driven by the difference in chemical potential^33^,^37^. A continuous growth of NPs is observed at the surface for *T*_A_ = 950 °C. As a result, the NP formation at the surface is attributed to atomic segregation, bulk and surface diffusion, and the nucleation with subsequent clustering, predominantly near grain boundaries.

To further clarify the formation and crystalline nature of the NPs at the surface, transmission electron microscopy (TEM) measurements were carried out on the BZO(0.02 and 0.06) films annealed at *T*_A_ = 950 °C. Figure 1b shows cross-sectional TEM images for the annealed BZO films. The thickness of the films was found to be 101 ± 12 and 152 ± 8 nm, respectively. These films consist of three distinct regions; (*i*) a NP-distributed surface, (*ii*) the bulk film, and (*iii*) an interfacial layer. By comparing these images, it can be seen that the density of NPs at the surface increases with increased Be concentration in the annealed BZO films. Scanning TEM (STEM) measurements were performed to obtain the compositional distribution in the annealed BZO films by atomic contrast (*Z*-contrast)^38^. The upper image of Fig. 1c shows dark field STEM for the BZO(0.06) film annealed at *T*_A_ = 950 °C. A pronounced contrast is observed between the surface and the bulk film due to the higher accumulation of lighter Be at the surface relative to the larger Zn. The darker spots are distributed across the main film with increasing density toward the surface. These directly indicate out-diffusion and local segregation of Be towards the surface during high-temperature annealing. It was also found that the NPs were pinned at the surface, as shown in the lower TEM image of Fig. 1c. To evaluate the crystalline feature of the surface NPs, high-resolution TEM and Fast-Fourier transform (FFT) were performed (Fig. 1d). The filtered FFT images correspond to the red squares I and II in the NP and adjacent layers in the film, indicate that both crystalline phases have *c*-axis wurtzite structures. The lattice parameters for the NP were determined to be *a* = 2.63 Å and *c* = 4.27 Å based on the spacing of the (0001) and 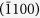 planes, while those for the surface layers of the film were *a* = 3.28 Å and *c* = 5.24 Å, respectively. The lattice parameters of the NP are similar to that of hexagonal BeO (*a* = 2.69 Å and *c* = 4.37 Å)^39^. In addition, the lattice parameters of the film underneath are close to those of bulk ZnO (*a* = 3.25 Å and *c* = 5.20 Å). These results suggest that the surface NPs are mainly composed of Be and O forming BeO crystals resulting from the out-diffusion of Be in the bulk towards the surface (see Supplementary Fig. 1).

### Be-concentration-dependent film thickness and thermal dissociation with *T*_A_

**Figure 2:**
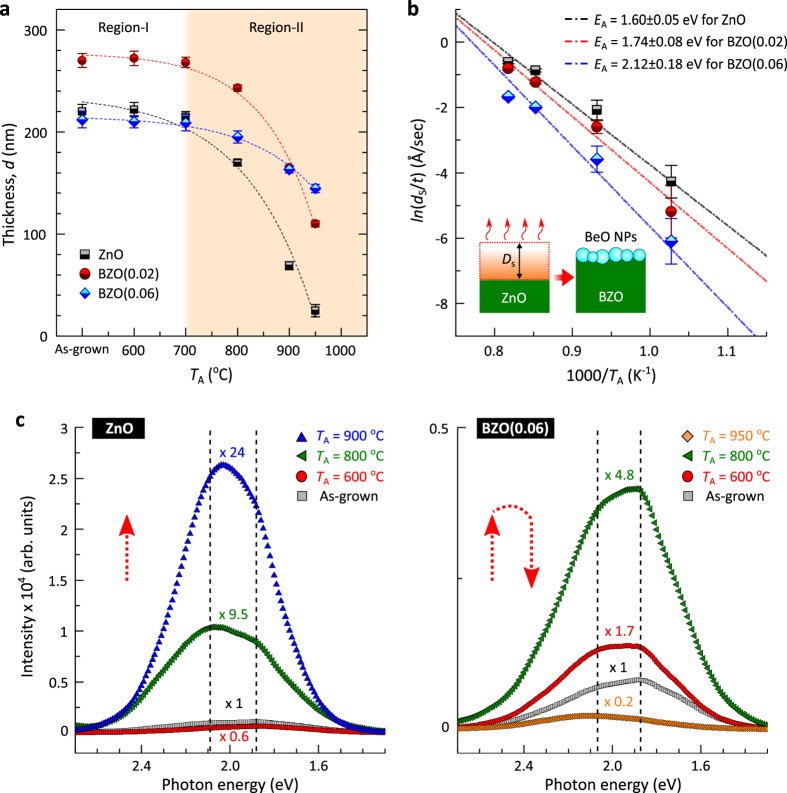
Thermal lattice dissociation and the associated deep band emissions in BZO films. (**a**) Variations in the thickness of the undoped ZnO and BZO(0.02 and 0.06) films as a function of *T*_A_. (**b**) Thermal dissociation rates with different *T*_A_ for ZnO [black-filled squares], BZO(0.02) [red-filled circles], and BZO(0.06) [blue-filled lozenges]. Data points are derived from *D*_s_/t, where *D*_s_ and t are the sublimated thickness of the films and annealing time, respectively. Dash-dot lines represent the Arrhenius plots for thermal dissociation rates of the films with corresponding activation energies, *E*_A_. (**c**) Low temperature (12 K) PL spectra for deep level emissions in the as-grown and annealed ZnO (*T*_A_ = 600, 800, and 900 °C) and BZO(0.06) films (*T*_A_ = 600, 800, and 950 °C).

Thermal resistance measured as a function of the thickness of the ZnO and BZO films was examined as a function of *T*_A_ by performing infrared (IR) reflectance and simulation^40^. The thicknesses and the dissociation rates [the sublimated thickness of the films (*D*_s_)/annealing time (*t*)] of the films with different *T*_A_, obtained from the modelling, are presented in Table 1. Figure 2a shows the thickness variations of the ZnO and BZO(0.02 and 0.06) films with different *T*_A_. This is divided into region-I, *T*_A_ ≤ 700 °C, and region-II, *T*_A_ ≥ 700 °C. No significant change in the film thickness was found in region-I. By contrast, in region-II, the thickness of the undoped ZnO films decreased considerably as *T*_A_ was increased up to 950 °C. The rate of thickness decrease in the BZO alloy films was lowered by increasing the Be concentration. This is indicative of Be-induced reduction in the thermal decomposition of the alloy films. Figure 2b shows Arrhenius plots of the dissociation rate of the films with respect to inverse annealing temperature, 1000/*T*_A_; all values were deduced as a function of *T*_A_. The dissociation activation energies, *E*_A_, of the films were extracted from the decomposition kinetics by applying a zero-order rate law in a single direction, *D*_s_/*t* = *C*_A_exp(−*E*_A_/*k*_B_*T*_A_), where *C*_A_ is the Arrhenius pre-exponential factor and *k*_B_, the Boltzmann constant, and the Arrhenius kinetic parameter, *E*_A_, is proportional to the enthalpy of reaction^41^,^42^. The of *E*_A_ in the BZO films increased up to 2.12 ± 0.18 eV with increasing estimated values of *E*_A_ from the decomposition reaction rate of the undoped ZnO film is 1.60 ± 0.05 eV, which is close to the Zn-O bond strength of 1.64 eV/molecule^43^. Hence, reduction of the film thickness is directly proportional to the amount of thermal dissociation of the ZnO lattice during annealing. The value of *E*_A_ in the BZO films increased up to 2.12 ± 0.18 eV with increasing Be concentration. This increase results from two correlated factors: (*i*) the effective coverage of atomic diffusion pathways (*e*.*g*. surface and grain boundary areas) by nucleated/grown BeO NPs preventing the thermal evaporation of dilute atoms and (*ii*) stronger bonding energy of BeO than that of ZnO^44^.

The high-temperature lattice dissociation and atomic diffusion/sublimation were expected to produce lattice defects in the annealed films. Photoluminescence (PL) measurements performed at 12 K show deep level emissions in the as grown and annealed ZnO and BZO(0.06) films (*T*_A_ = 600, 800, and 950 °C) as shown in Fig. 2c. Two predominant deep band emission (DBE) components were found in both the ZnO and BZO films around 2.09 and 1.88 eV, respectively. Amid the controversy of defect-associated DBE in ZnO, we assign these two bands to Zn vacancy (V_Zn_)-related defect emissions; considering the above atomic dissociation processes at high temperatures, and the subsequent formation of V_Zn_ clusters in the films. No strong emission was observed around 2.4 eV, attributed to oxygen vacancies (V_O_) from any of the samples^45^. Similar emission features have also been observed in ion-implanted and annealed ZnO^46^,^47^,^48^. Thus, the two DBEs could be responsible for optical transitions from the ZnO/BZO conduction bands (residual shallow donors such as Zn interstitials) to energy states of the vacancy cluster defects below by ≈2.1 eV (≈1.9 eV). The intensity of the defect-related DBE in the ZnO films is considerably increased (by > × 10) for temperatures up to *T*_A_ = 900 °C. This reveals that high-temperature annealing facilitates the formation of V_Zn_ clusters, corroborating the thermal dissociation and diffusion data together with the thickness reduction in the films. On the other hand, the DBE intensity in the annealed BZO film increases up to *T*_A_ = 800 °C (lower intensity compared to that of ZnO), then, falls remarkably at *T*_A_ = 950 °C. Such a turn-over in the population of V_Zn_ has been addressed by counter-diffusion of cations, *i.e.*, outward (inward) diffusion of Be (Zn), and the effective compensation of vacancy defects in the fully-transformed BZO alloy films^40^.

### Nucleation and growth of BeO NPs at the surface of the annealed BZO alloy films

The observations of thermally-induced recrystallization of the metastable BZO alloy films show that annealing causes a phase transformation in the alloy in conjunction with lattice strain relaxation and a redistribution of the constituent atoms^40^. This corresponds to a shrinkage of the band gap energy in the bulk back towards that of undoped ZnO and an enrichment of the Be concentration at the surface. Such a phase transformations in a highly mismatched alloy system is typically due to the solubility limit and low thermal stability of Be under a miscibility gap, leading to a minimization of the total free energy. During the annealing process, significant outward diffusion of Be occurs from the bulk to the surface together with the dissociation of ZnO bonds. Hence, the constituent atoms, Zn, Be, and O, diffuse at the surface and along grain boundaries that are effective pathways for atomic transport. Since the vapour pressure of Be is much lower than that of Zn, the out-diffusing Be predominantly accumulates at the surface and grain boundaries, while the dissociated Zn evaporates. This leads to the increase of Be concentration at the surface, reaching critical supersaturation and giving rise to the secondary phase nucleation.

**Figure 3:**
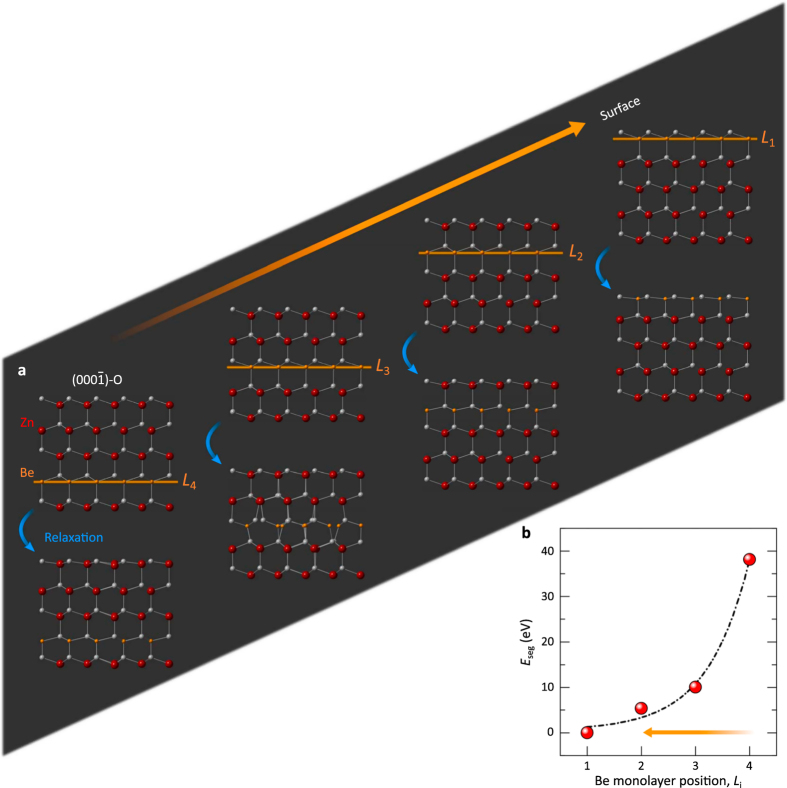
Model representing Be segregation at the surface. (**a**) Side views modelled for four different atomic configurations with different full-Be monolayer positions (*L*_4_ − *L*_1_) in O-terminated ZnO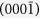 slab supercells. Upper (lower) models represent as-constructed slab supercells (relaxed supercells). (**b**) The Be segregation energy profile calculated for four different atomic configurations corresponding to the position (*L*_*i*=1−4_) of the Be monolayer within an O-terminated surface of the wurtzite ZnO slab.

To simulate the energetics of Be out-diffusion, DFT calculations were performed modelling ZnO slabs with repeated substitution of Be into all Zn sites across single cation monolayers from the uppermost surface, *L*_1_, down to the inner layers; four cation-monolayers were taken into account, *L*_1_ − *L*_4_ (Fig. 3a). The Be segregation energies calculated by *E*_seg_ = *E*(*L*_*i*=1−4_) − *E*(*L*_1_) show a significant increase when the substitute Be monolayer takes positions in lower Zn monolayer as illustrated in Fig. 3b. The tendency toward Be segregation at the surface corresponds to the thermodynamic stabilization of the alloy films, which is in good agreements with our experimental observation of Be transport toward the surface (Supplementary Information, Fig. 3). Since the free energy, *E*^F^, for the formation of BeO (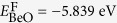 at 0 K) is much lower than that of ZnO (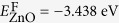 at 0 K)^40^, the diffused/segregated Be atoms preferentially react with thermally dissociated/diffused oxygen forming the energetically favourable BeO phase. These theoretical results corroborate the out-diffusion of Be and the formation of secondary phase BeO NPs at the surface of the annealed BZO films.

In classical nucleation theory (CNT)^26^,^30^,^31^, the kinetics of the BeO nucleation reaction is primarily described by: (*i*) the mobility of the Be and O atoms for structural attachments, which is significantly enhanced with temperature, and (*ii*) the total Gibbs free energy change, Δ*G*(*R*_n_), for the formation of the nuclei^31^,^49^. Assuming a spherical shape for the BeO crystals within the parent BZO phase, Δ*G*(*R*_n_) is given by


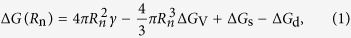


where *R*_n_ is the radius of the nucleus and *γ* is the surface tension (related to the free energy change for the formation of a solid BeO surface). The free energy change per unit volume, Δ*G*_V_, for the formation of the BeO nucleus dependent on the temperature (*T*) and solute supersaturation, *S*, according to Δ*G*_V_ = *k*_B_*Tln*(*S*)/*v*_m_, where *v*_m_ is the molar volume of the bulk crystal. Here, Δ*G*_s_ is a finite misfit strain energy per unit volume proportional to the volume of the inclusion, and Δ*G*_d_, the energy of pre-existing defects in the volume, means that some free energy is released through the destruction of the defects by the creation of the nucleus. The critical free energy of the stable BeO nucleus, Δ*G*_c_, with respect to *R* yields an equilibrium size, *R*_c_, from the relation, *d* Δ*G*(*R*)/*dR* = 0. The critical radius, *R*_c_, represents the minimum size of a stable BeO nuclei that allows them to endure and continue to grow: Values below *R*_c_ would lead to dissolvation of the nuclei. Hence, nucleated BeO particles need to surpass a thermodynamic energy barrier 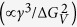 for the growth of secondary phase nuclei.

In the classical approach to particle growth derived from Lifshitz, Slyozov, and Wagner (LSW) theory under low supersaturation and quasi-steady-state limit, the driving force of OR is a decrease in the solubility of particles with increased radius, *R*_g_. The growth of larger particles is at the expense of smaller dissolving particles due to the high solubility and surface energy of the smaller ones^50^,^51^. Furthermore, the surface reaction-limited particle growth normally gives rise to an asymmetric particle size distribution. Therefore, in most NP growth processes, nucleation and growth concurrently occur throughout particle formation and hence the final particles show a broad size distribution (as can be seen in Fig. 1a).

**Figure 4:**
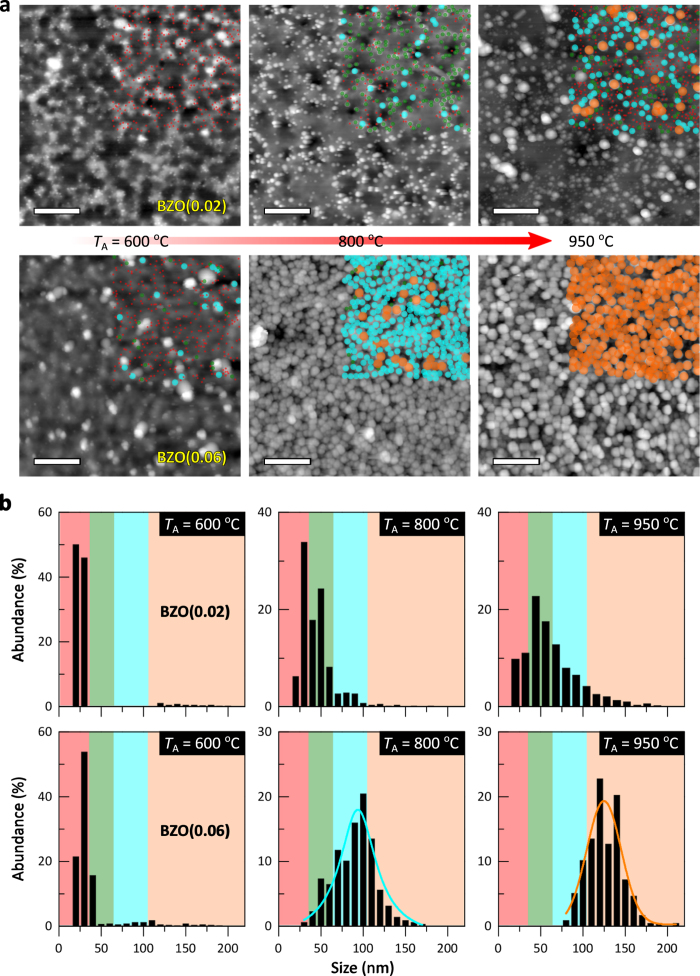
AFM images of the growth of self-assembled NPs and the associated particle size distribution. (**a**) Topographic AFM images (3 × 3 μm^2^) for the surface morphology of the BZO(0.02) [upper panels] and BZO(0.06) [lower panels] films annealed at *T*_A_ = 600, 800, and 950 °C. Scale bar, 600 nm. The size difference of the surface NPs in the top-right square area of each image are differentiated by color: red dots correspond to a size of ≈30 nm; green dots, ≈50 nm; blue dots, ≈100 nm; orange dots, ≈120 nm. (**b**) The relative particle size distributions for the grown NPs at the surface of the annealed alloy films.

Accordingly, using the nucleation-and-growth kinetic theory for NPs, the growth characteristics and the associated size distribution of the secondary phase BeO NPs originated from the thermally induced phase transformation of the BZO films are examined. Figure 4a shows AFM images (3 × 3 μm^2^) of the temperature-dependent (*T*_A_ = 600 − 950 °C) variations in the growth and size distribution of the BeO NPs at the surface of the annealed BZO films (*x* = 0.02 and 0.06). The images show a gradual growth of the particles at the surface of the alloy films with increasing *T*_A_. More interestingly, distinct size distributions of the grown NPs are observed in the annealed films with different initial Be compositions (Fig. 4b). The particle-size-distribution (PSD) of the NPs is broadened at the surface of low Be content alloy films as it displays a gradual growth of the particles at the surface of the films with *T*_A_. This is associated with the typical ripening effect in a transient growth regime as mentioned above. By contrast, the narrowing of the PSD together with increasing the average NP size are seen for the alloy films with high Be composition. This suggests that different NP growth characteristics induced by varied growth kinetic factors such as the supersaturation level for different Be concentrations at the surface as a function of *T*_A_ (see Supplementary Information 2).

**Figure 5:**
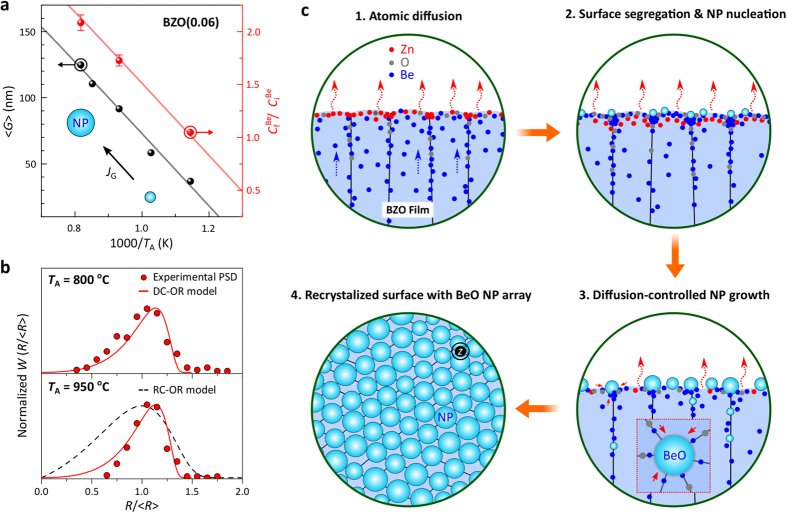
Limited particle growth characteristic of NP self-assembly. (**a**) Variations in the average size, 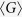, of the BeO NPs and Be concentration ratio, 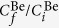, at the surface of the annealed BZO(0.06) film as a function of reciprocal annealing temperature (1000/*T*_A_). (**b**) Stationary size distribution, *W*, of the NPs for two high-temperature-annealed alloy films as a function of particle radius ratio, 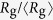, where 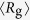 is the average radius of the NPs. The limited PSDs are predicted from the associated size distribution function within the LSW theory for diffusion-controlled (solid line: DC-OR) and reaction-controlled (dashed line: RC-OR) Ostwald ripening. (**c**) Schematic representations of thermally driven out-diffusion of the constituent atoms, and the nucleation-and-growth processes of the secondary phase BeO NPs within the diffusion-controlled OR growth mode in a transformed BZO alloy film. This growth mode is enforced by the high Be supersaturation level at the surface together with the additional/continuous injection of Be during the growth process, resulting in a focusing of the NP size distribution.

To interpret the distinct NP growth features observed with different surface Be concentrations, we address a correlation between the growth mode and PDS of the BeO NPs in the annealed BZO(0.06) films. As can be seen in Fig. 5a, the mean size, 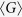, of the BeO NPs is increased monotonically from ≈37 nm at *T*_A_ = 600 °C to ≈125 nm at *T*_A_ = 950 °C. This is accompanied by a linear increase in Be concentration and narrowing of the PSD at the surface. The change in Be concentration ratio in Fig. 5a is defined by 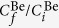, where 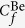 and 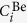 are the measured Be concentration of the annealed samples in the steady-state regimes and the initial concentration of the as-grown samples, respectively. This reveals that the BeO NP growth rate, *J*_G_, primarily depends on the degree of Be solute supersaturation at the surface. Therefore, Be acts to be growth-rate-controlling assuming that no concentration gradient exists in the oxygen for the diffusion process of Be and O atoms into BeO precipitates of radius, *R*, within the steady-state approximation^52^.

In the LaMer mechanism^50^,^53^, the nucleation and growth stages for the formation of NPs are mainly dependent on two mechanisms: (*i*) diffusion-controlled growth and (*ii*) reaction-controlled growth. In the former, all monomers participate in the nucleation processes and thus the mean NP size is dependent on *S*, while in the latter, the mechanism is particle-size-dependent (the curvature-induced surface reaction of the particles). However, if the particle growth takes place in the transient growth regime before either OR or the equilibrium between the particles and monomers occurs, then the focusing of PSD is possible to continue in the diffusion-controlled growth mode by modifying the kinetics of the reactions (a steep drop of growth temperature or hot injection of participates or additional/continuous injection of participates). This would lead to inhibition of the nucleation of new NPs and a period of the broadening and growth. These effective approaches maintain the simultaneous diffusion-controlled particle growth and the resultant narrowing of PSD, and have been widely applied for the monodispersive formation of various colloidal particles^30^,^37^,^54^. In this study, given a substantial amounts of Be out-diffused from the bulk (*i.e.* Be supersaturation) allows continuous participation in the growing of NPs. In this model, the continuous injection of Be solute predominantly favours the focusing of the PSD of the BeO NPs with a larger mean size until the completion of the growth process, after which, the samples were cooled to room temperature. In Fig. 5b, such BeO NPs were formed and characterized for the annealed BZO(0.06) films at *T*_A_ = 800 and 950 °C. This is performed by applying the stationary size distribution function in the diffusion-controlled OR (DC -OR) based on the LSW theory that^37^,^55^


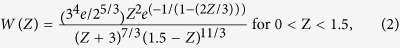



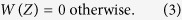


Here, 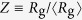 and 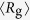 is the average particle radius; the cut-off is at *Z* = 1.5. The size focusing of the secondary phase BeO NP growth in the high-temperature annealed alloy films is predominantly associated with high Be solute saturation and simultaneous inclusion of the newly arrived Be solutes from the bulk within the diffusion-controlled growth mode. The overall nucleation and growth processes of the secondary phase BeO NPs at the surface of the thermally-transformed BZO films are schematically presented in Fig. 5c.

### Passivation effects of surface NPs on chemisorption

**Figure 6:**
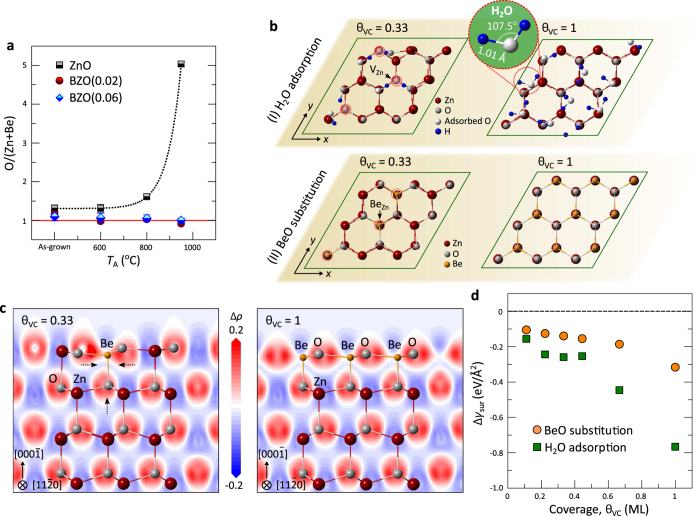
Surface relaxation by H_2_O adsorption and BeO substitution. (**a**) Profiles of O to (Zn + Be) ratio for the as-grown and annealed ZnO and BZO(0.02 and 0.06) films at different *T*_A_. The elemental ratios were obtained from composition analysis by fitting the XPS spectra. (**b**) Upper panel I: relaxed ZnO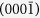-O surface structures with coverages, *θ*_VC_ = 0.33 and 1 of Zn-O VCs at the topmost ZnO double layer. Dissociated H_2_O, *i.e.*, H and OH, replace V_Zn_ and V_O_ sites, respectively. For dissociative H_2_O adsorption, surface stabilization favours the formation of ionic O-H bonds adjacent to V_Zn_ at *θ*_VC_ ≤ 0.44, while the dominant formation of individual H_2_O molecules occurs at *θ*_VC_ ≥ 0.67. Lower panel II: relaxed ZnO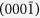-O surface structures with the same coverages at the topmost ZnO double layer. Be and O replace V_Zn_ and V_O_ sites, respectively. (**c**) Contour plots of charge-density difference, Δ*ρ* (|*e*|/Å^3^), along 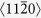 planes for the final atomic configuration of the BeO-substituted ZnO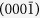-O surfaces with different coverage, *θ*_VC_ = 0.33 and 1. Blue and red regions represent electron depletion and accumulation, respectively. (**d**) Variations in the corresponding surface energy, Δ*γ*_sub_, of ZnO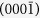 as a function of the coverage, *θ*_VC_, for both dissociated H_2_O adsorption and BeO substitution.

The ratio of O to Zn + Be, [O/(Zn + Be)], at the surface of the undoped ZnO and BZO(0.02 and 0.06) films for different *T*_A_ has been obtained by fitting the XPS core level spectra, as depicted in Fig. 6a. The ratio of O anions to Zn and Be cations for the as-grown ZnO and alloy films was found to be around 1.2 to 1.33, respectively. The O-richness of the as-grown films is attributed to the stabilization on polar ZnO surfaces by surface atomic reconstructions and surface chemisorption. The latter includes dissociative water species (H, O-H, H_2_O) and other environmental oxide species such as CO_2_ depending on the polarity of the surface according to electron counting rules^56^,^57^. A considerable increase in the ratio of O/Zn in the annealed ZnO films was observed increasing from 1.33 to 5.14 up to *T*_A_ = 950 °C. Since, during high-temperature annealing, lattice defects and defect clusters as V_Zn_ and V_O_ are produced. The increased defect density at the surface gives rise to an increase in the hydrophilicity and wettability at the surface of these oxides^58^,^59^,^60^. From the fitting of the XPS O 1*s* core level spectra for the annealed ZnO films (Supplementary Information, Fig. 4), surface-chemisorption-related components such as hydroxides and water molecules at *E*_B_ ≈ 532 − 533 eV to the Zn-O bond (*E*_B_ ≈ 530 eV) was substantially increased for *T*_A_ = 950 °C. Such an increase in the O to Zn ratio is associated with the evaporation of Zn and O atoms and the subsequent increase in thermally-induced surface defects. By contrast, the ratio O/(Zn + Be) of the BZO films reduced to unity and remained constant, indicating that increasing Be concentration at the surface of the alloy films lowers and ultimately stop further the chemical adsorption (Supplementary Information, Fig. 5). This reveals that thermally created lattice vacancy-defects at the surface are compensated by atomic substitution, namely, V_Zn_ + Be_out-diff_ → Be_Zn_.

To gain further insight into this compensation process (adsorptions of oxygen species and atomic substitutions) leading to the stabilization of the defective polar ZnO surfaces, appropriate models of ZnO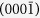 surface structures for DFT calculations were prepared as shown in Fig. 6b (details are shown in Methods and Supplementary Information 6 & 7.). As high-temperature annealing causes dissociation of a number of Zn-O bonds, which results in the thickness reduction of the undoped ZnO thin films, the Zn-O vacancy clusters (VCs) are expected to form at the surface^45^. Hence, different numbers, *N* = 1–9, of VCs were considered in the modelling of the ZnO slabs. The VCs (V_Zn−O_) are compensated separately by two independent processes of dissociative H_2_O adsorptions and BeO substitutions. Dissociated H^+^ (OH^−^) replaces V_Zn_ (V_O_) sites for H_2_O adsorptions^59^,^61^, while Be^2+^ (O^−2^) replaces V_Zn_ (V_O_) sites for BeO substitutions. These adsorption and substitution mechanisms are based on the balance between energy gained through the saturation of the surface broken bonds by dissociated water and BeO, and the energy cost for the splitting of water molecules and the substitution of BeO. The energy gain needs to be larger than the energy cost, leading to surface stabilization.

For relaxation of the adsorbing surfaces at low coverages (*θ*_VC_ ≤ 0.44) of VCs, dissociated H atoms diffuse and couple with the topmost O dangling bonds adjacent to V_Zn_ due to their electrostatic attraction, leading to the dominant formation of O-H bonds (Fig. 6b and Supplementary Information, Fig. 6). At high coverages (*θ*_VC_ ≥ 0.67), the dissociated O-H and H tend to form individual H_2_O molecules on the surfaces. In the case of BeO substitutions, the local atomic bond length at the surface reduces due to the local impact of the shorter Be-O bonds until the coverage of VCs by BeO reaches *θ*_VC_ = 0.33 (Supplementary Information, Fig. 8). The surface atomic rearrangement subsequently saturates along the wurtzite crystal symmetry in the high coverage regime (*θ*_VC_ ≥ 0.44) reaching an equilibrium lateral bond distance of *d* = 1.85 Å and a *c*-bond length, *d*_z_ = 1.79 Å for the topmost surface double layer (Supplementary Fig. S9). As seen in Fig. 6c, strong Be-O bonds are formed at the top layer, leading to charge accumulation along the ligands. These atomic and charge redistributions on the BeO substitution are caused by the electronegativity and atomic size difference between the Zn and Be with respect to O. The more ionic bonding character of the substituted BeO is responsible for the strengthening of the defective surface. Furthermore, the surface energy change, Δ*γ*_sub_, determined as a function of the coverage, *θ*_VC_, of H_2_O/BeO on VCs clearly show a continuous decrease in Δ*γ*_sub_ as the number of VCs increases for both H_2_O adsorptions and BeO substitutions (Fig. 6d). This implies that atomic vacancy defects and/or vacancy clusters facilitate the surface wettability of highly defective ZnO to lower the surface free energy. In the case of the transformed BZO alloys, water adsorption at vacant sites on the surface is effectively restrained by BeO substitution, before exposing the surface defects to the atmosphere. The successful formation of thermally robust BeO NPs suppresses both the thermal sublimation of ZnO at the surface for high *T*_A_ and defect-mediated chemical adsorption.

### Electrical characteristics of the annealed BZO films compared with that of the ZnO films

**Figure 7:**
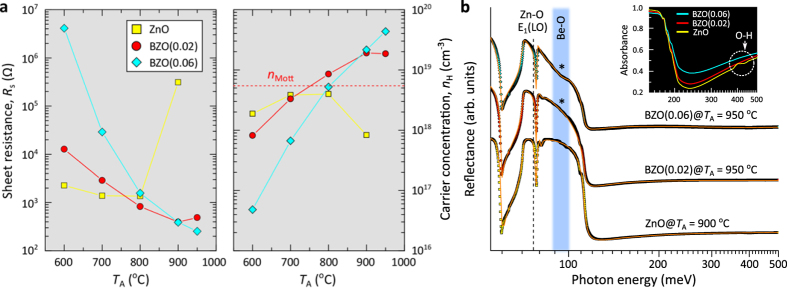
Electrical and optical characterization of the undoped ZnO and BZO films as a function of *T*_A_. (**a**) Room temperature sheet resistance and carrier concentration of the ZnO and BZO(0.02 and 0.06) films annealed at different *T*_A_. (**b**) Experimental IR reflectance plotted with the simulated spectra (orange lines) for the annealed ZnO (*T*_A_ = 900 °C) and BZO (*T*_A_ = 950 °C) films. An impurity mode (*) in the IR reflectance spectra of the BZO films is found at ~96 meV that is associated with BeO NPs. The adsorption peaks between 400 and 450 meV in the inset of (**b**) are due to surface O-H species.

Hall effect measurements were performed to investigate the effect of annealing on the electrical properties of the annealed ZnO and BZO(0.02 and 0.06) films as a function of *T*_A_. Figure 7a shows room temperature (RT) sheet resistance (*R*_*s*_) and Hall carrier concentration of the ZnO and BZO films annealed at different *T*_A_. The *R*_*s*_ decreases to 1.38 kΩ for the ZnO film annealed at 800 °C. This decrease is primarily attributed to an increase in the carrier mobility due to the thermally driven grain growth in the film. The ZnO film annealed at *T*_A_ = 900 °C exhibits a higher *R*_*s*_ ≈ 17 kΩ, (a lower Hall carrier concentration, *n*_H_ = 1.8 × 10^18^ cm^−3^ at RT) and the film annealed at *T*_A_ = 950 °C shows a highly insulating behaviour. This is due to high-temperature-induced lattice dissociations (Supplementary Information Fig. 10), the subsequent increase of compensating lattice defects such as V_Zn_, and surface effects, which are associated with coupling of grain boundaries and/or surface lattice defects and the related trap defects such as OH and O_2_. In contrast, a continuous lowering (increasing) of *R*_*s*_, up to 252 Ω, (carrier concentration, *n*_H_ = 4.3 × 10^19^ cm^−3^ at RT) was seen in the BZO(0.06) films annealed at *T*_A_ = 950 °C. The carrier concentration in the high-temperature (*T*_A_ ≥ 800 °C) annealed BZO films is above the Mott-transition concentration of ZnO, *n*_Mott_ = 5.7 × 10^18^ cm^−3^.

To get further insights into the electrical behaviour of the films, IR reflectance measurements and simulation of the annealed ZnO (*T*_A_ = 900 °C) and BZO (*T*_A_ = 950 °C) films were performed and the results (Fig. 7b) identify an impurity mode (~96 meV) in the BZO films, which is related to Be-O phonon mode^40^. In addition, absorption peaks at 400–450 meV associated with surface O-H species were observed only in the annealed ZnO films as shown in the inset of Fig. 7b. Such oxygen species usually act as electron traps and build potential barriers at the surface defects, which cause charge compensation and a strong impedance opposing charge carrier transport at the surface of many oxides^4^,^12^,^14^,^15^. Comparison with the electrical properties of the annealed ZnO films suggests that BeO NPs in the annealed BZO films prevent the adsorption of acceptor-like oxygen species by covering the surface lattice and planar defects, while annealing increases *n*-type donor sources such as V_O_ or Zn_i_^15^,^40^. This leads to a decrease (increase) in *R*_*s*_ (*n*_H_) of the BZO films annealed at high temperatures which eventually forms highly conductive film layers with significant reduction in charge compensation.

## Discussion

In this study, the spontaneous coverage and growth of secondary phase BeO NPs effectively enhances both the thermal-oxidation resistance and the durability of the active *n*-type ZnO layers within the phase-transformed BZO. The NPs prevent the formation of extrinsic acceptor surface states by inhibiting the physiochemical interactions between the induced lattice defects or planar defects and environmental oxygen species such as H_2_O/OH^−^ and O_2_/O^2−^. This leads to a reduction of the associated charge compensation and significantly improves charge carrier concentration and carrier transport properties of the oxide material. Therefore, controlling the nucleation-and-growth and particle-size distribution of environmentally inert secondary phase NPs by thermodynamic phase transformation and solid-state reactions in metastable oxide alloys is key for effective passivation of reactive oxide surface. This novel phase transformation concurrently driven by strain relaxation and defect-mediated atomic compensation is also applicable to numerous metastable material systems subject to the structural interiors and material composition. Additionally, from a technological perspective, this facile method offers not only a cost-effective process for the fabrication of environmentally robust oxide materials, but also provides a guideline to the design of future oxide optoelectronic applications obviating the need for post-surface-protective treatments.

## Methods

### Film growth and thermal treatments

Undoped ZnO and BZO(0.02 & 0.06) films were grown on Al_2_O_3_(0001) substrates by RF co-sputtering. Oxygen partial pressure was 2 × 10^−2^ mbar (based pressure = 1.8 × 10^−8^ mbar) and growth temperature was 500 °C. Change in Be concentration of the BZO films were controlled by adjusting RF power to the Be metal target (99.9%) from 30 to 40 W, while the RF power of the ZnO ceramic target (99.999%) was fixed at 100 W. Prior to the film growth, surface contaminants of all the targets were removed using an RF power setting of 50 W. All the as-grown films were thermally treated at various temperatures between 600 °C and 950 °C for 60 min in a continuous flow of N_2_ gas. All annealing temperatures were attained at a ramping rate of ≈20 °C/min.

### Characterisation

Structural determination of the films were carried out by high-resolution and powder X-ray diffraction using a Panalytical X’Pert Pro MRD and a Panalytical X’Pert Pro MPD, respectively, with an incident beam hybrid monochromator giving pure Cu K_*α*1_ radiation (*λ* = 1.5406 Å). The optical absorption of the films was measured at normal incidence using a Perkin-Elmer Lambda 25 UV/V is spectrometer. Surface morphology was studied by tapping-mode AFM measurements. XPS measurements were performed in ultra-high vacuum (UHV: base pressure = 3 × 10^−11^ mbar) using an Omicron SPHERA hemispherical analyzer and a monochromatic Al K_*α*_ X-ray source (*hv* = 1486.6 eV). For surface sensitive measurements, the emitted photoelectrons from the surface of the films were collected with variable take-off angle (TOA, *θ*_TOA_), 30° ≤ *θ*_TOA_ ≤ 90°. During the XPS measurements, surface charging effects were compensated using a low energy electron flood gun (Omicron CN10). The *E*_B_ scale was calibrated to the C 1*s* core-level peak with the overall energy resolution of 0.47 eV. The XPS spectra were fitted using a Shirley background subtraction and Voigt line shapes. Compositional ratios of the elements (Zn, O, Be and C) in the films were determined after correcting for the inelastic mean free path of photo-emitted electrons via the TPP-2M formula^62^ and applying Scofield photoionized cross-sections^63^. Far- and Mid-IR reflectance spectra at an incident angle of 11° with respect to the surface normal were recorded in the range of 50 − 8000 cm^−1^ using a Bruker Vertex 70v Fourier transform infrared (FTIR) spectrometer. The thickness of the films was determined by simulating the reflectance spectra in a stratified medium with associated complex dielectric functions. The IR reflectance simulations were carried out utilizing the classical Drude model and factorized model considering the longitudinal-optical phonon-plasmon coupling, impurity modes, and the anharmonic effects in the complex dielectric function of the films^40^. Field emission transmission electron microscopy (FETEM, a Tecnai G^2^ F30 S-Twin) was used with an acceleration voltage of 300 kV to characterize the microstructural properties of the annealed BZO samples at *T*_A_ = 950 °C. Defect distribution in the ZnO and BZO films were plotted as a function of *T*_A_ by variable temperature (T = 12 − 300 K) photoluminescence measurements using a He-Cd laser (*λ* = 325 nm). A complementary long-pass optical filter with a cut-off wave length of 420 nm was used to increase the dynamic range of deep level emissions in the samples and remove the laser line and its second order diffraction. All of the PL measurements were performed using a constant optimized exposure for the absolute comparison of the luminescence intensity from the samples. The electrical properties of the films were characterized by performing variable temperature (T = 5 − 300 K) Hall effect measurements in the van der Pauw configuration. Indium solder was used to make ohmic contacts to all of the etched corners of the 5 × 5 mm^2^ samples.

### Computational methods

First-principle calculations were performed based on density functional theory (DFT) within the local density approximation using the Cambridge Sequential Total Energy Package (CASTEP)^64^,^65^. A plane-wave basis set with a kinetic energy cut-off of 400 eV was employed and the electron-ion interactions were formulated using ultrasoft pseudopotentials. After geometric optimization of the unit cell, the equilibrium lattice parameters for wurtzite ZnO were obtained to be *a* = 3.185 Å and *c* = 5.149 Å, respectively. The vacuum spacing of 20 Å was introduced in the z-direction to prevent inter-slab interactions. The surface was modelled by eight ZnO double layers allowing structural relaxation within a slab supercell of the four uppermost double layers. All calculations were carried out using 3 × 3 supercells composed of approximately 150 atoms. Numerical integrations over the Brillouin zone were performed only at the Γ-point due to the large size of the supercells. The atomic positions in all the supercells were continually relaxed to the residual forces below than 0.05 eV/Å. In the modelled ZnO slab supercells, any charge transfer from the uppermost O layer to the lowest Zn layer induced by the net dipole moment and electrostatic field across the slab was corrected by altering the valency of Zn atoms on the lowest layer to +1.5^57^,^66^. Other surface stabilization processes accompanied by atomic reconstructions (atomic removals and/or the formation of islands) or additional H^+^ and OH^−^ adsorption on the residual surface atoms were ruled out. The chemical adsorption energy, *E*_ads_, (atomic substitution energy, *E*_sub_) at the surface, namely, the energy released during chemisorption (substitution) corresponds to the binding energies of V_Zn−O_ and H_2_O (BeO). For quantitative evaluation, the average energy values are defined as


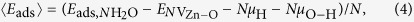



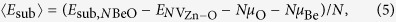



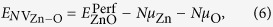


where 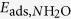 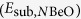 is the total energy of the ZnO slab supercells that contains *N* number of adsorbed H_2_O (substituted BeO). 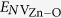 is the total energy of the ZnO slab supercells with the *N* number of 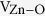, 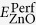 is the total energy of the ZnO supercell without any vacancies. *μ* defines the associated chemical potential. The average adsorption (substitution) energy gain per H_2_O (BeO) is related to the change in the surface energy, Δ*γ*_*sub*_, via


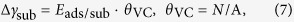


where *A* is the surface area.

### Data Availability

All data created during this research are provided in the results section of this paper and Supplementary Information.

## Additional Information

**How to cite this article**: Park, D.-S. *et al.* Surface passivation of semiconducting oxides by self-assembled nanoparticles. *Sci. Rep.* **6**, 18449; doi: 10.1038/srep18449 (2016).

## Supplementary Material

Supplementary Information

## Figures and Tables

**Table 1:** The thicknesses, *D*, and thermal dissociation ratio, *D*_s_/*t*, of ZnO, BZO(0.02), and BZO(0.06) films as a function of *T*_A_.

*T*_A_ (°C)	ZnO		BZO (0.02)		BZO (0.06)	
	*D*(nm)	*D*_s_/*t* (Å/sec)	*D* (nm)	*D*_s_/*t* (Å/sec)	*D* (nm)	*D*_s_/*t* (Å/sec)
As-grown	220 ± 7	…	270 ± 7	…	212 ± 8	…
600	222 ± 7	…	272 ± 7	…	209 ± 6	…
700	215 ± 5	0.014	268 ± 5	0.005	208 ± 6	0.005
800	170 ± 3	0.125	243 ± 5	0.083	195 ± 4	0.034
900	69 ± 4	0.419	165 ± 2	0.292	160 ± 2	0.147
950	25 ± 3	0.542	110 ± 3	0.444	114 ± 3	0.205
