# Supplementary material for: Surface passivation of semiconducting oxides by self-assembled nanoparticles
Source: Sci Rep. 2016 Jan 13;6:18449. doi: 10.1038/srep18449 (PMC4725940; doi:10.1038/srep18449)
Supplement: Supplementary Information [file srep18449-s1.pdf]

Supplementary Information: Surface passivation of semiconducting oxides by self-assembled nanoparticles

Dae-Sung Park, Haiyuan Wang, Sepehr K. Vasheghani Farahani, Marc Walker, A. Bhatnagar, Djelloul Seghier, Chel-Jong Choi, Jie-Hun Kang, Chris F. McConville*

Supplementary Information 1

XRD θ-2θ profiles of the as-grown ZnO and BZO(0.02 and 0.06) films on Al_2O_3(0001) substrates are shown in Supplementary Fig. 1a. The diffracted (0002) peaks shift towards higher angles from 34.11° to 34.42° as the Be concentration increases. This reveals a decrease in the *c*-axis lattice constant due to incorporation of smaller Be atoms into the host ZnO lattice. All of the diffracted peak positions for the (0002) plane of the as-grown films were at lower 2θ angles, compared to that of ZnO bulk (2θ = 34.42°). These diffraction features are associated primarily by two factors, (*i*) strain effects (*i.e.* a compressive strain of the in-plane lattice of the film) for the as-grown alloy film on the highly mismatched Al_2O_3(0001) substrate, and (*ii*) a small Be composition (≤ 6 %), as shown in the inset of Supplementary Fig. 1a. Such high biaxial lattice strain usually gives rise to a predominant expansion of the *c*-axis lattice parameter, particularly for strained films far away from the bulk ZnO parameters relative to the Be-induced lattice shrinkage. Furthermore, evidence of phase separation with a splitting of the (0002) diffraction peak was observed as the Be concentration increased to 6 %. This results from the local Be composition in the alloy films and a minimization of the total energy in the metastable alloys, *e.g.*, elastic energy, interfacial energy, and surface energy[1-4], and the formation of a multi-phase crystallinity of the alloy film with compositional inhomogeneity. Hence, the Be composition in the as-grown alloy films was determined and compared with different compositions of x = 0.02 and ≈ 0.06 by applying Vegard's law[5], as shown in Supplementary Fig. 1a.

The diffraction patterns of the annealed alloy films with different T_A (600 – 950 °C) are shown in Supplementary Fig. 1b. All (0002) peaks shift to higher angles as T_A increases and

reaches at 2θ = 34.42° of the bulk ZnO(0002) value. The multiple diffraction peaks of the as-grown BZO(0.06) film tend to be a single peak at $T_A \geq 700$ °C. These results clearly indicate that annealing induces strain relaxation and Be redistribution in these metastable alloy films on highly mismatched substrates. Extra diffraction peaks in both annealed films appear around 2θ = 41.0° at $T_A \geq 800$ °C. These peaks correspond to the wurtzite BeO(0002) indicative of the thermally-driven nucleation-and-growth of BeO nanoparticles (NPs) in the transformed alloy films.

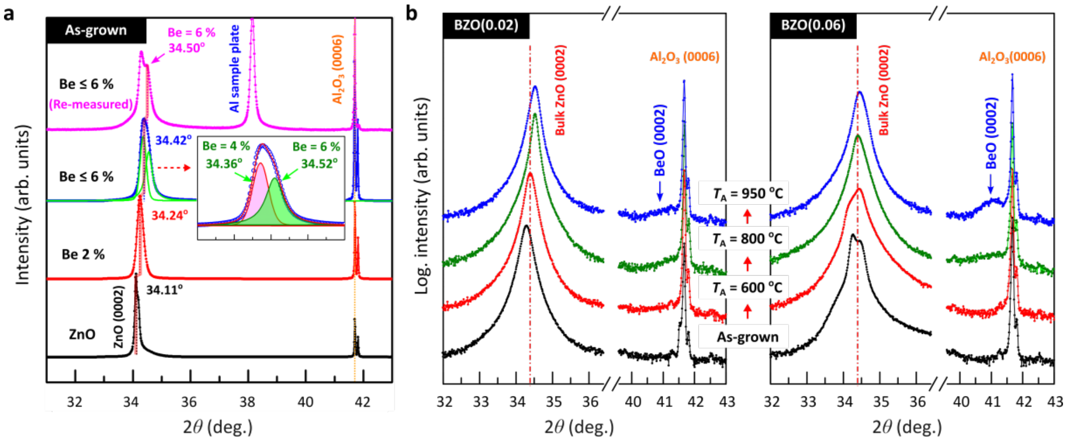

Supplementary Figure 1. XRD θ-2θ patterns for (a) the as-grown ZnO and BZO(0.02 and 0.06) films and for (b) the as-grown and annealed BZO films on Al_2O_3(0001) as a function of T_A.

Supplementary Information 2

The effects of thermal annealing on the optical absorption properties of the BZO(0.02 and 0.06) films was examined and compared to those of undoped ZnO. All of the as-grown and annealed films are highly transparent above 80 % in UV and visible range as shown in Fig. 2a. The absorption coefficient, α, of the films was calculated using optical transmittance spectra and Fig. 2b shows α^2 as a function of photon energy for different Be concentrations and annealing temperature (T_A). The absorption edge is determined by linear extrapolation of the sharp onset to the horizontal portion of the spectra for the as-grown films and shifts to higher energies with Be composition. In addition, an increase in the tailing of the spectra is observed due to band gap widening and structural deterioration induced by the incorporation of Be in the host ZnO lattice (see Supplementary Fig. 1a). Significant changes in the position and tailing of the optical absorption edge in both the ZnO and BZO films are also observed with increasing T_A. The absorption edge (optical band gap energy) of the as-grown and annealed ZnO films, increases up to T_A = 800 °C, while the energy decreases with further increase in $T_A \geq 900$ °C (Supplementary Fig. 2c). The former is due to an increase in thermally-induced donor-like point defects, a reduction in the carrier-trap centers (*e.g.* charged structural defects or grain boundaries), and subsequent conduction band-filling effects in the annealed undoped films[6]. This corresponds to an increase in the carrier concentration in the films, which were determined by Hall effect measurements (see Fig. 7a in the main text). The latter for $T_A \geq 900$ °C primarily arises from thermal decomposition and the resulting structural deterioration of the ZnO lattice. This compensates the conduction electrons through the introduction of deep acceptor levels in the band gap, together with tailing effects. The red-shift of the optical band gap leads to insulating behavior in the high-temperature-annealed ZnO films ($T_A \geq 900$ °C) and in turn no optical response was found at T_A = 950 °C. On the other hand, a continuous decrease in the optical band gap energy of both BZO films was found with T_A mainly due to loss of Be from the film bulk as a result of thermal redistribution and lattice strain relaxation (presented in our previous work[7].). However, it should be noted that the optical values obtained from the annealed

alloy films are affected by many-body interactions, namely, electron-electron and electron-phonon interactions, which result in band gap renormalization[8].

Figure 2d shows the normalized XPS spectra for the Zn 3*s* and Be 1*s* core levels for the BZO(0.02 and 0.06) films as a function of T_A. The spectral position of the Be 1*s* peak at a binding energy (E_B) of 114.3 eV indicates Be-O chemical bonding as a result of Be incorporation into the host ZnO[9]. The Be 1*s* peak intensity for both alloy films gradually increases with T_A up to 950 ◦C. The atomic concentration of Be at the surface of the films was determined as a function of T_A by quantitative analysis of the intensity of the O 1*s*, Zn 3*s*, and Be 1*s* core level peaks taking into account the inelastic mean free path of photo-excited electrons and relative sensitivity factor for each element in the medium[11]. Figure 2e shows an increase in the ratio of Be to Zn, Be/(Zn+Be), from 0.10 [0.24] to 0.27 [0.51] for BZO(0.02) [BZO(0.06)] respectively as T_A is increased up to 950 °C. It is worthy of note that the initial Be composition at the surface of the as-grown BZO films is higher than that for the bulk film (Supplementary Information 1), due to surface segregation/reconstruction and the removal of constituent atoms on the polar surface to reduce the surface energy[10]. Consequently, these opposing trends, the decrease in the optical band gap energy and the increase in the surface Be concentration with increasing T_A, are indicative of the out-diffusion of Be from the bulk to the surface, resulting in a lower Be concentration in the bulk.

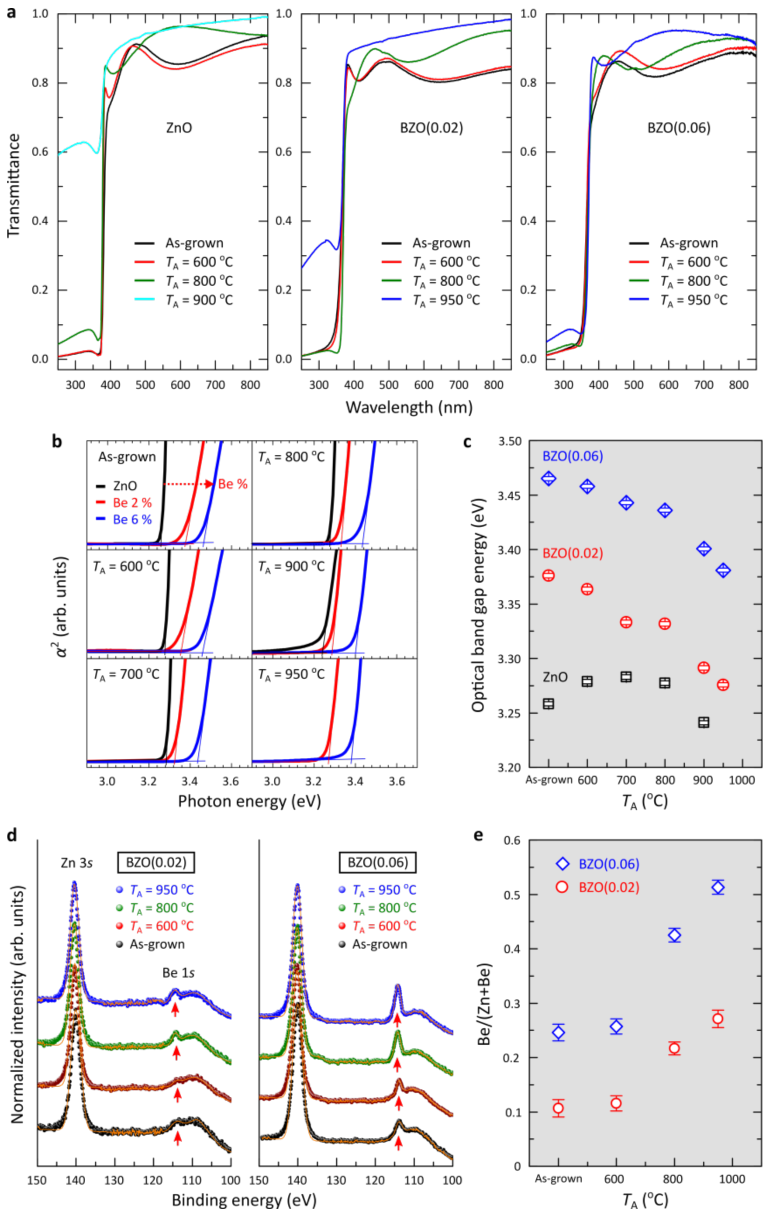

Supplementary Figure 2. (a) Transmittance spectra for the as-grown and annealed ZnO, BZO(0.02), and BZO(0.06) films. (b) The square of the absorption coefficient, α^2 as a function of photon energy obtained from the transmittance spectra of the ZnO and BZO (0.02 and 0.06) films with different T_A. (c) Comparison of the optical band gap energy of the films as a function of T_A. (d) Normalized XPS spectra of the Zn 3s and Be 1s core levels for the as-grown and annealed ZnO and BZO films at different T_A. All the spectra were collected at normal emission and normalized for the same Zn 3s peak intensity. The red-arrows correspond to the binding energy of Be-O bond around E_B $\approx$ 114 eV. (e) The profiles of Be to Zn ratio, Be/(Zn+Be), at the surface of the films with T_A.

Supplementary Information 3

Angle-dependent XPS measurements were carried out to evaluate the distribution of BeO nanoparticles (NPs) at the surfaces of the BZO (0.02 and 0.06) films annealed at T_A = 950 °C. The XPS spectra were collected by varying the emission angle, θ_{TOA}, of the photoelectrons within the XPS probing geometry (see insert of Supplementary Fig. 3a) to increase the XPS surface sensitivity. Figure 3a shows the normalized XPS Zn 3*s* and Be 1*s* core level spectra for the annealed BZO films. The Be 1*s* peak intensity increases with decreasing θ_{TOA}, indicating the upward concentration gradient of Be conducive to the formation of surface NPs. After XPS quantitative analysis, the concentration gradient of Be at the surfaces was determined to range from 27 % [51 %] to 49 % [66 %] for the annealed BZO(0.02) [BZO(0.06)] as TOA decreased from θ_{TOA} = 90° to 30° (Supplementary Fig. 3b). In order to determine the depth-dependent Be composition at the surface, the inelastic mean free path, IMFP (λ), for the Be 1*s* core level spectra of Al K_α XPS in approximation of the different density of the surface area with varying Be concentration were calculated by using the TPP-2M equation; different densities of surface region due to varying Be concentrations were considered in this approximation[11]. The depth profile of the Be concentration in the NPs was attained using $\lambda_{eff} = \lambda \sin\theta$ (nm), where λ_{eff} (effective IMFP) and θ_{TOA} are variable parameters, respectively, as shown in the inset of Supplementary Fig. 3b. These results provide direct evidence of significant accumulation of BeO NPs at the surface of the transformed alloy films.

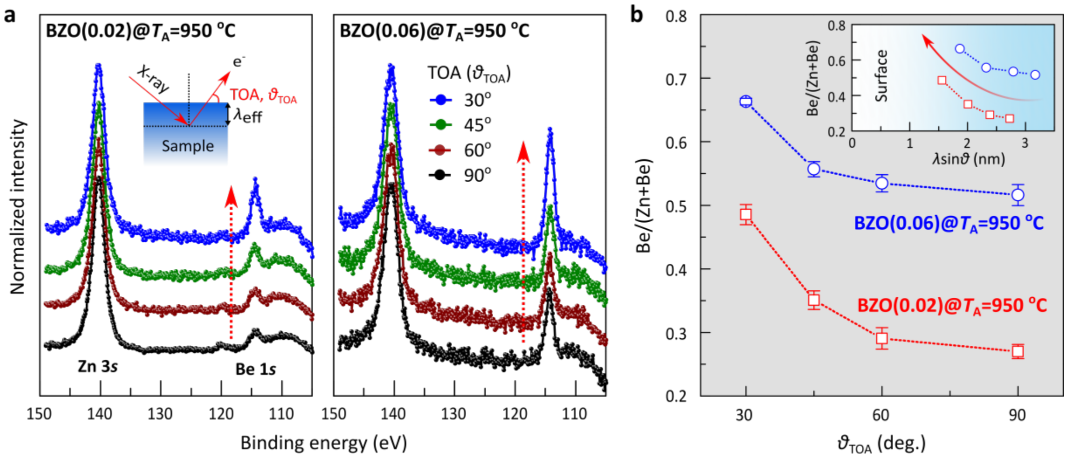

Supplementary Figure 3. (a) XPS spectra of Zn 3*s* and Be 1*s* core levels with varying θ_{TOA} from 90° to 30° for the BZO(0.02 and 0.06) films annealed at T_A = 950 °C, and normalized to have the same Zn 3*s* peak intensity. The insert in the left panel of Fig. 3a is a schematic of the X-ray photoemission geometry. (b) The TOA dependence of the surface Be to Zn ratio, Be/(Zn+Be), for the annealed BZO films. The inset shows the depth profile of the surface Be to Zn ratio. The TPP-2M formula was employed to calculate the effective inelastic mean free path, λ_{eff}, for the photo-emitted electrons from Be 1*s* by Al K_{α} excitation energy using the approximated density of the BZO medium.

Supplementary Information 4

Angle-dependent XPS O 1*s* spectra for the ZnO films annealed at T_A = 600, 800, and 950 °C are shown in Supplementary Fig. 4a. From the fitting of the XPS spectra, the three convoluted components were assigned to Zn-O bond in the wurtzite ZnO lattice at E_B = 529.7 - 530.1 eV; Zn-O-H bond from surface hydroxides at E_B = 531.5 - 532.0 eV; and other surface water molecules/carbonate species at E_B = 533.2 - 533.8 eV. The latter two components are from chemisorbed oxygen at the surface of ZnO. The intensity of Zn-O-H peak increased significantly relative to that of Zn-O with increasing T_A up to 950 °C. This indicates that high temperature annealing induced a substantial increase in surface chemisorption. By decreasing the TOA of the photo-emitted electrons from θ_{TOA} = 90° to 30°, a higher ratio of Zn-O-H/Zn-O bond states was found at the surface of all the annealed films (T_A = 600 °C and 950 °C) as shown in Supplementary Fig. 4b. This ratio increased from 8.62 (θ_{TOA} = 90°) to 9.45 (θ_{TOA} = 30°) for T_A = 950 °C as a result of an increase in the high-temperature-induced surface defect density to be replaced by adsorbates. These observations provide direct evidence to the increasing lattice dissociation toward the topmost surface of the undoped ZnO films with increasing T_A, causing an increase in surface defects and thickness reduction. Quantitative XPS analysis has been performed to calculate the distribution of surface atoms as a function of T_A.

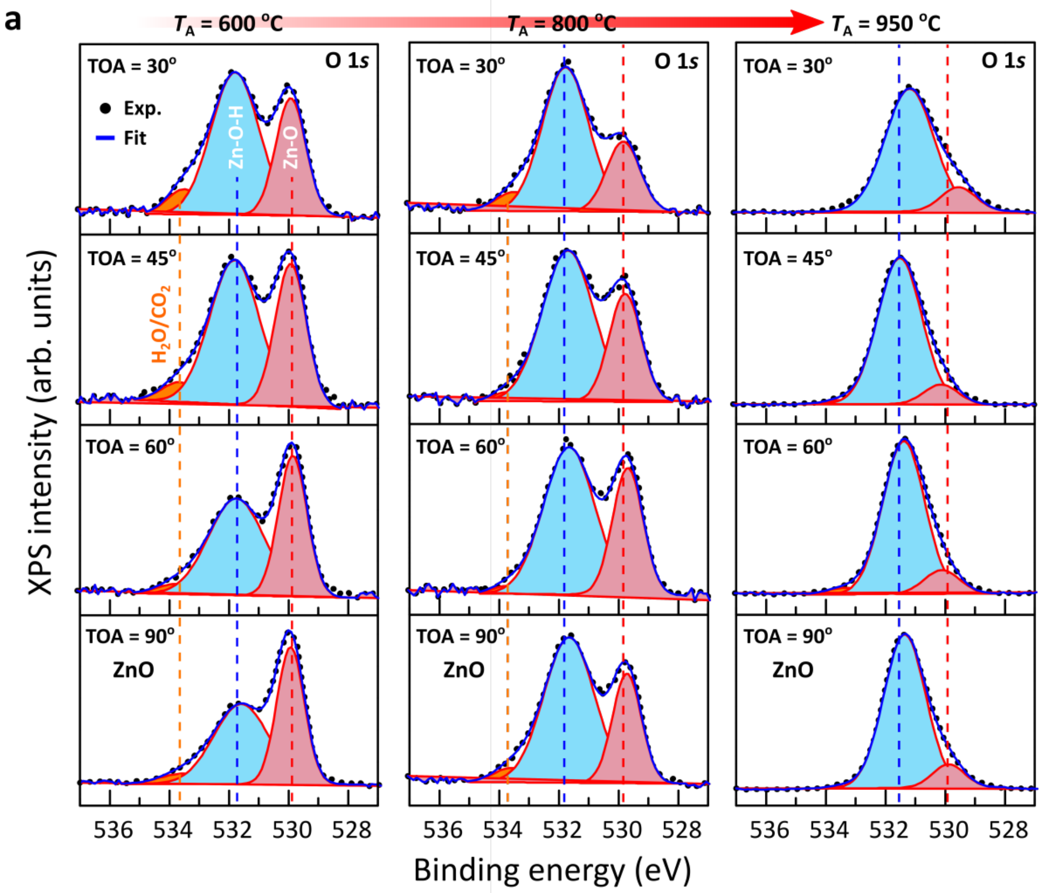

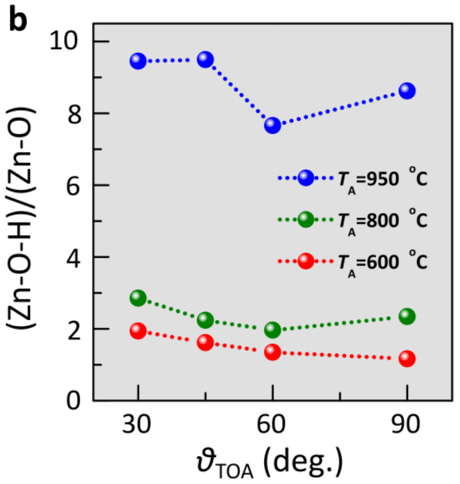

Supplementary Figure 4. (a) XPS O 1*s* core level spectra for the annealed ZnO films at T_A = 600, 800, and 950 °C as a function of TOA, θ_{TOA} = 30° - 90°. These core level spectra were deconvoluted into three distinct components, which are assigned to Zn-O, Zn-O-H, and other chemisorbed oxygen species, H_2O/CO_2, respectively. Details of the fitting parameters are presented in Table 1. (b) The ratio of Zn-O-H to Zn-O bond states in the annealed films as a function of θ_{TOA}.

Supplementary Table 1. XPS fitting parameters [E_B, Lorentzian percentage of the Voigt line shape (L), and full width at half maximum (FWHM)] of the O 1*s* core level spectra for the annealed ZnO films at T_A = 600, 800, and 950 °C.

T_A (°C)	Bonding	E_B (eV)	L (%)	FWHM (eV)
As-grown	Zn-O	529.85±0.05	8	1.26±0.05
	Zn-O-H	531.32±0.04	8	1.75±0.07
600	Zn-O	529.91±0.07	8	1.17±0.07
	Zn-O-H	531.79±0.07	8	2.06±0.15
	H_2O/CO_2	533.62±0.2	8	1.48±0.29
800	Zn-O	529.73±0.05	8	1.22±0.09
	Zn-O-H	531.67±0.03	8	1.97±0.06
	H_2O/CO_2	533.61±0.02	8	1.24±0.21
950	Zn-O	529.89±0.25	8	1.33±0.07
	Zn-O-H	531.35±0.16	8	1.74±0.09
	H_2O/CO_2	533.2±0.1	8	1.32±0.25

Supplementary Information 5

Figure 5 shows O 1*s* core level spectra at the surface of the high-temperature annealed BZO films, compared to the ZnO films. For the BZO(0.02 and 0.06) films, the peak area associated with Be-O bonds ($E_B \approx 531.1$ eV) is increased with T_A, which is consistent with the increase in peak area of the Be 1s as a result of thermally-induced out-diffusion of Be to the surface of the alloy films. There is no significant change with T_A in the higher E_B sides ($E_B \approx 532$ - 533 eV) pertaining to surface chemisorption. Hence, these results clearly support surface passivation of the annealed alloy films by BeO NPs as opposed to defect-mediated chemisorption, which is not observed at the surface of the annealed ZnO films.

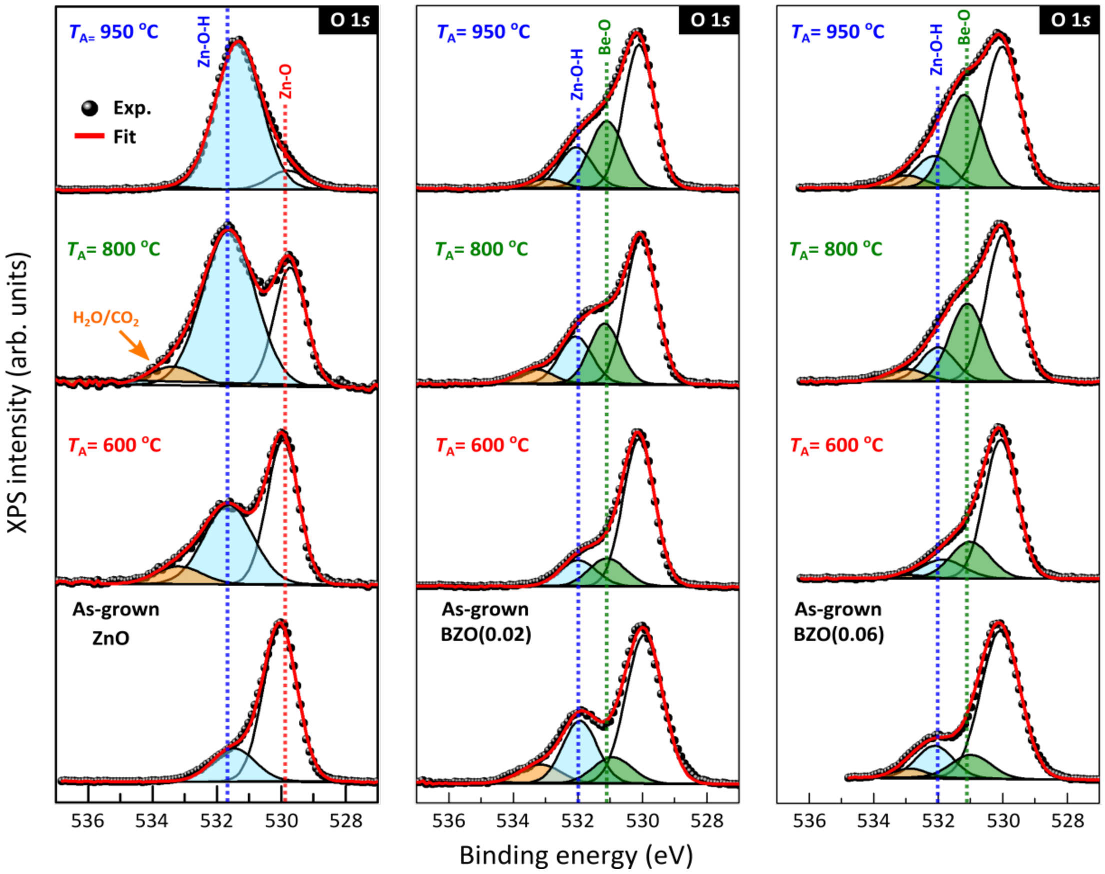

Supplementary Figure 5. XPS O 1*s* core level spectra of the undoped ZnO and BZO(0.02 and 0.06) films with different T_A (600, 800, and 950 °C). All XPS spectra were collected at normal emission (θ_{TOA} = 90°). Four distinct components were considered in the fitting of the BZO spectra due to additional Be-O bonding.

Supplementary Information 6

DFT calculations were performed to investigate the surface relaxation mechanism on the defected ZnO slab supercells by half-dissociative adsorption with respect to the number of Zn-O vacacny clusters (VCs). Figure 6a shows that the half-dissociated OH and H from each water molecule (H_2O) replace V_O and V_{Zn}, respectively, forming O-H_O (blue circles) and H_{Zn} (red circles) on a Zn-O VC. The surface is relaxed by the rearrangement of the O-H and H: (i) H end of O-H tilts by 45° with respect to surface normal toward the V_{Zn} and (ii) H atom moves to bond with the nearest-neighbor O. These rearrangements were relaxed by the formation of two O-H bonds hexagonally facing the V_{Zn} on their H ends (Supplementary Fig. 6b). The newly formed O-H bond can be driven by the electrostatic attraction (repulsion) between the positively charged H and negatively charged O (tilted $O^{\sigma'-}$-$H^{\sigma+}$, where $\sigma' > \sigma$). In this manner, the half-filled orbital of the surface O atoms adjacent to the V_{Zn}, become occupied. Identical formation behavior of the V_{Zn}-mediated O-H bonds was also observed at the low coverage of dissociated water molecules (up to 4-VC, $\theta_{VC} \leq 0.44$). For high coverage ($\theta_{VC} \geq 0.67$) of VCs, dissociated H and O-H tend to form individual water molecules. Hence, water-molecular adsorption increases the adsorption energy at the surface as illustrated in Supplementary Fig. 7. This arises from partially-filled dangling bonds of the surface O atoms by H_2O adsorption as the ionic bonding in H_2O is stronger than that of the dissociated H_2O with the surface atoms. These results indicate that vacancy cluster defects at ZnO surface mediate the surface reactions of dissociated O-H and H with changes made in their bonding mechanisms (including type of bonds, angles, strain, *etc.*) depending on VC coverages.

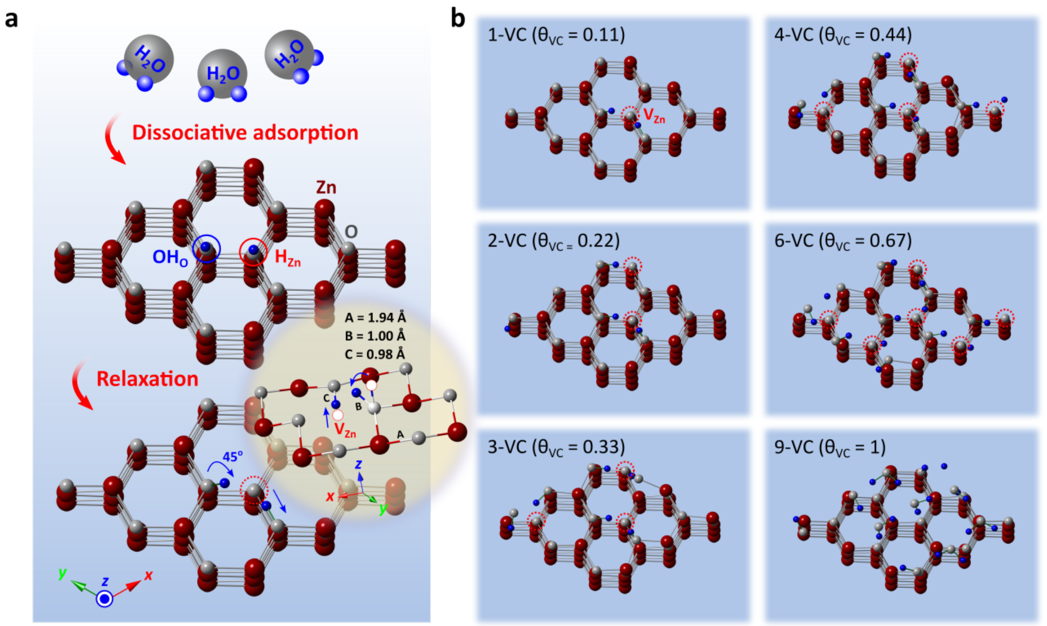

Supplementary Figure 6. (a) A schematic representation for the formation mechanism of O-H bonds on a defected ZnO surface through dissociative adsorption and relaxation of water molecules (half-dissociated OH and H) on a Zn-O VC. (b) Top view of relaxed ZnO slab (3 × 3) supercells modeled as a function of the number of VCs (1-VC to 9-VCs) replaced by decomposed H_2O molecules. The positions of VC sites were randomly selected on the topmost double-layer of the slab supercells for DFT calculations.

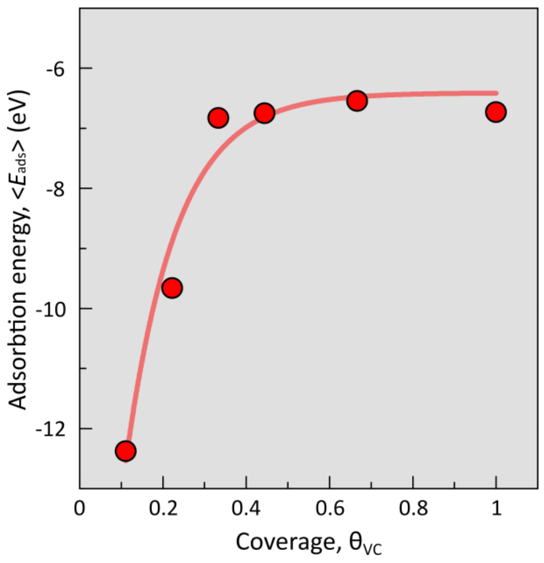

Supplementary Figure 7. The average adsorption energy of dissociated O-H and H calculated with respect to the coverage of vacancy clusters, θ_{VC}, at the surface of ZnO.

Supplementary Information 7

The bond length of the three laterally coordinated O atoms tends towards that of bulk BeO ($d_{\mathrm{Bulk\,BeO}}$ = 1.67 Å) through Be substitution until θ_{VC} = 0.33 (Supplementary Fig. 8). Increasing the coverage of VCs up to θ_{VC} = 1 leads to the dilatation of lateral bonds, reaching an equilibrium bond length of d = 1.85 Å maintaining the wurtzite symmetry. By contrast, Be-O bond length monotonically decreased along the z-direction $[000\bar{1}]$ to d_z = 1.79 Å as shown in Supplementary Fig. 9.

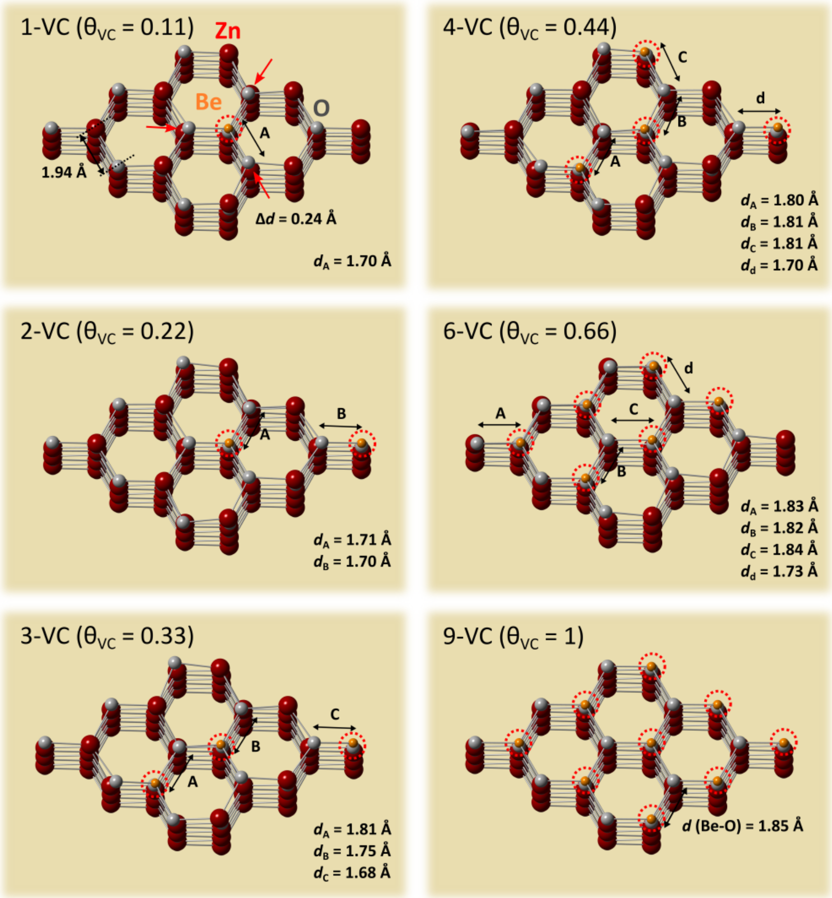

Supplementary Figure 8. Top view of relaxed ZnO slab (3 × 3) supercells modeled as a function of the number of VCs (1-9) being substituted by BeO. Be and O atoms replace V_{Zn} (cyan-dot circles) and V_O, respectively. The positions of VC sites were randomly selected on the topmost double-layer of the slab supercells for the DFT calculations. All surfaces were relaxed by BeO substitutions and the subsequent rearrangements of the surrounding Zn and O atoms.

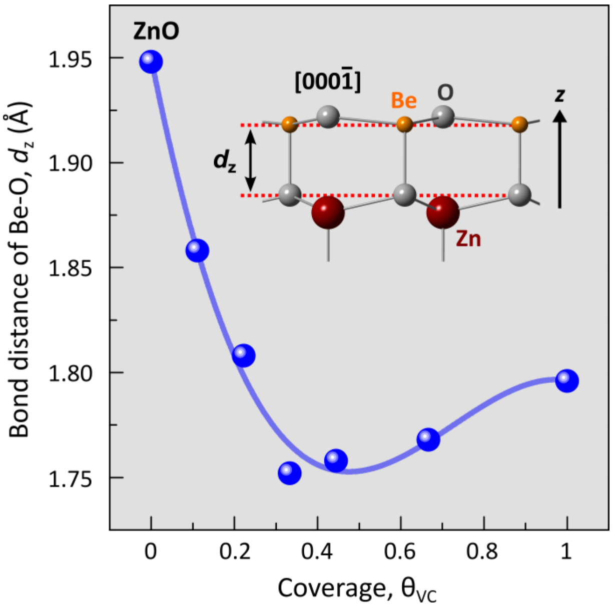

Supplementary Figure 9. (a) Variations in the average bond distance, d_z, between the topmost Be cations and underneath O atoms in the relaxed ZnO(000$\bar{1}$) slab supercells as a function of the coverage of VCs being substituted by BeO.

Supplementary Information 8

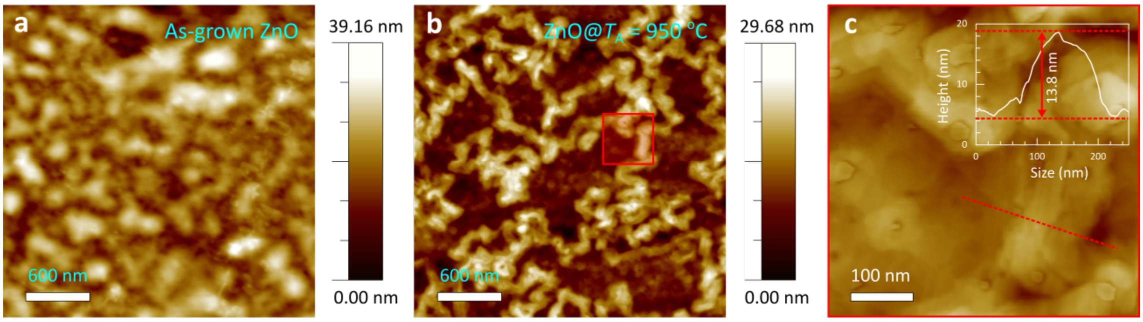

Supplementary Figure 10. AFM topography (3 × 3 μm^2) images for (a) the as-grown and (b) annealed ZnO films at T_A = 950 ∘C. (c) AFM topography (0.5 × 0.5 μm^2) image corresponds to the red square area in the AFM topography (3 × 3 μm^2) image for the surface morphologies of the annealed ZnO.

Supplementary References

1. Park, D.-S., Krupski, A., Sanchez, A. M., Choi, C.-J., Yi, M.-S., Lee, H.-H., McMitchell, S. R. C. & McConville, C. F. Optimal growth and thermal stability of crystalline $Be_{0.25}Zn_{0.75}O$ alloy films on Al_2O_3(0001). *Appl. Phys. Lett.* **104**, 141902−141906 (2014).
2. Es-Souni, M. Computer simulation of segregation kinetics in ternary alloys. *Scr. Metall. Mater.* **23**, 919−924 (1989).
3. Duxbury, N., Bangert, U., Dawson, P., Thrush, E. J., Van der Stricht, W., Jacobs, K. & Moerman, I. Indium segregation in InGaN quantum-well structures. *Appl. Phys. Lett.* **76**, 1600−1602 (2000).
4. Bogusławski, P. & Bernholc, J. Surface segregation of Ge at SiGe(100) by concerted exchange pathway. *Phys. Rev. Lett.* **88**, 166101−166104 (2002).
5. Denton, A. R. & Ashcroft, N. W. Vegard's Law. *Phys. Rev. A* **43**, 3161−3164 (1991).
6. Fujiwara, H. & Kondo, M. Effects of carrier concentration on the dielectric function of ZnO:Ga and In_2O_3:Sn studied by spectroscopic ellipsometry: analysis of free-carrier and band-edge absorption. *Phys. Rev. B* **71**, 075109–075118 (2005).
7. Park, D.-S., Vasheghani Farahani, S. K., Walker, M., Mudd, J. J., Wang, H., Krupski, A., Thorsteinsson, E. B., Seghier, D., Choi, C.-J., Youn, C. J. & McConville, C. F. Re-crystallization of highly-mismatched $Be_xZn_{1-x}O$ alloys: formation of a degenerate interface. *ACS Appl. Mater. Interfaces* **6**, 18758–18768 (2014).
8. Roth, A. P., Webb, J. B. & D. F. Williams Band-gap narrowing in heavily defect-doped ZnO *Phys. Rev. B* **25**, 7836–7839 (1982).
9. Park, D.-S., Mudd, J. J., Walker, M., Seghier, D., Krupski, A., Saniee, N. F., Choi, C.-J., Youn, C. J., McMitchell, S. R. C. & McConville, C. F. Pinning effect on the band gap modulation of crystalline $Be_xZn_{1-x}O$ alloy films grown on Al_2O_3(0001). *CrystEngComm.* **16**, 2136–2143 (2014).
10. Dulub, O., Diebold, U. & Kresse, G. Novel stabilization mechanism on polar surfaces: ZnO(0001)-Zn. *Phys. Rev. Lett.* **90**, 016102–016105 (2003).
11. Hofmann, S. *Auger- and X-ray photoelectron spectroscopy in materials science* (Springer, 2013).
